# From Conversion to Resection for Unresectable Hepatocellular Carcinoma: A Review of the Latest Strategies

**DOI:** 10.3390/jcm12247665

**Published:** 2023-12-13

**Authors:** Chen Liang, Zhaoqian He, Qiang Tao, Xiang Tang, Lingmin Jiang, Xinyue Tu, Zonghao Liu, Hua Chen, Feihu Xie, Yun Zheng

**Affiliations:** 1 Department of Liver Surgery, Sun Yat-sen University Cancer Center, Guangzhou 510060, China; liangchen@sysucc.org.cn (C.L.); hezq1@sysucc.org.cn (Z.H.);; 2 State Key Laboratory of Oncology in South China and Collaborative Innovation Center for Cancer Medicine, Sun Yat-sen University Cancer Center, Guangzhou 510060, China

**Keywords:** hepatocellular carcinoma, unresectable liver cancer, conversion therapy

## Abstract

Hepatocellular carcinoma (HCC) is one of the most common malignant tumors in China, accounting for the majority of primary liver cancer cases. Liver resection is the preferred curative method for early-stage HCC. However, up to 80–85% of patients have already missed the opportunity of radical surgery due to tumor advances at the time of consultation. Conversion therapies are a series of medications and treatments for initially inoperable patients. For early-stage unresectable HCC (uHCC) patients, conversion therapies are designed to meet surgical requirements by increasing the volume of the residual liver. Meanwhile, for advanced cases, conversion therapies strive for tumor shrinkage and down-staging, creating the opportunity for liver resection or liver transplantation. This review summarizes the latest advances in conversion therapies and highlights their potential for improving the survival benefit of patients with uHCC.

## 1. Introduction

Primary liver cancer is currently the fourth most common cancer and the second leading cause of cancer-related death in China, of which hepatocellular carcinoma (HCC) accounts for 75–85% of the incidence [1]. For patients with HCC, surgical treatment remains an important method to achieve long-term survival. However, most patients with hepatocellular carcinoma have an insidious onset and often lose the opportunity for surgery at the time of diagnosis. Inoperable liver cancer can be divided into technically unresectable, including patients with intolerable systemic conditions and insufficient residual liver volume, and oncologically resectable, which is technically resectable but cannot obtain better results than non-surgical treatment [2]. Owing to the large scale of HCC cases and clinical practice, the China Liver Cancer Staging (CNLC) system was established and has been widely adopted in China and abroad [3]. According to the CNLC system, radical treatments such as surgical resection, local ablation, and liver transplantation are recommended for patients with stage I a(single nodule ≤5 cm) and I b(single nodule >5 cm, or 2–3 nodules ≤3 cm) and selected patients with II a(2–3 nodules >3 cm) [1]. This is in line with the latest 2022 edition of the BCLC strategy, which recommends radical ablation, resection and transplantation for patients with stage 0 and stage A(Single, or 2–3 nodules each ≤3 cm), and transplantation for selected patients with stage B (multinodular) who meet the expanded criteria for liver transplantation [4]. However, concerning inadequate compensatory liver volume and insufficient residual liver function after radical treatment, usually predicted by preoperative evaluation, some patients may experience insufficient surgical margins. Additionally, patients with poor overall conditions may not tolerate surgery. These cases are classified as technically unresectable HCC. 

Advanced HCC mainly includes patients with CNLC stage II b (≥4 nodules), III a(vascular invasion), and III b(extrahepatic metastases). Studies showed that surgical treatment does not result in better survival outcomes compared to systemic therapy for advanced cases [2]. Among them, cases at CNLC stage II b are suitable for non-surgical treatments, primarily transarterial chemoembolization (TACE). The majority of CNLC stage III a and III b cases are initially unresectable, and the preferred treatment approach is systemic therapy. These patients are classified as oncologically unresectable HCC.

In this review, we have explored the concept of conversion therapy and summarized the patterns and recent advances in the treatment of unresectable hepatocellular carcinoma.

## 2. Conversion Therapy for Technically uHCC: Making It Grow

Cirrhosis from various causes is the most important link in the process of hepatocellular carcinoma, and 85%~95% of patients with hepatocellular carcinoma have different degrees of cirrhotic background [5]. Post-hepatectomy liver failure (PHFL) is a potentially fatal complication following major liver resection. The indocyanine green (ICG) clearance test is one of the most widely used preoperative tests to assess the incidence of PHFL in all patients, including those with mild fibrosis and advanced fibrosis. Patients undergoing liver resection should meet certain criteria, including an indocyanine green retention rate at 15 min (ICG-R15) of less than 30% and a residual liver volume (RLV) above 30–40% of the standard liver volume (SLV) [1]. The ratio of RLV to SLV correlates with postoperative outcomes, especially with PHLF. The formula for calculating SLT is based on a linear correlation between total liver volume and body weight or body surface area (BSA), based on a large patient population or autopsy data. In addition, Stéphanie et al., found that the simplified RLV/body weight ratio (RLVBWR) accurately assesses the functional limits of hepatic resection, as well as the RLV-SLV, but in a simpler manner [6]. In cases of impaired liver function, a larger portion of functional liver tissue should be preserved. The main reason that limits surgical intervention in patients with surgically unresectable liver cancer is insufficient compensatory liver function after liver resection. Therefore, making the residual liver volume grow is an important approach in the conversion therapy of technically unresectable liver cancer.

### Portal Vein Embolization and Associating Liver Partition and Portal Vein Ligation for Staged Hepatectomy (ALPPS)

Portal vein embolization (PVE) was first reported by Makuuchi et al., in 1982 [7]. In patients with insufficient FLR, embolization was performed by embolizing the portal vein branches near the tumor before surgery to promote enlargement of the remnant liver. However, due to the long waiting time for PVE to induce residual liver hypertrophy, there is a risk of further loss of surgical opportunity due to tumor progression.

German scholar Schlitt first proposed the two-stage liver resection technique known as associating liver partition and portal vein ligation for staged hepatectomy (ALPPS) in 2007. The first implementation of ALPPS was carried out by Schnitzbauer et al., in 2012 [8]. Since then, it has been widely used in the resection of colorectal liver metastases. The procedure follows two steps: in the first step, the liver is mobilized and the portal vein is ligated. It takes two to four weeks to let the liver compensate. And the tumor-containing liver lobe is resected after the remaining liver volume meets the requirements at step two. A randomized controlled study comparing portal vein embolization (PVE) and ALPPS in patients with liver cancer and hepatitis B-related cirrhosis showed that ALPPS patients achieved a higher surgical resection rate (97.8%) and were able to increase future liver remnant (FLR) volume more rapidly than PVE, both in the short term and long term, with comparable benefits [9]. However, in the presence of liver cirrhosis, the functional recovery of newly regenerated liver cells may be delayed. Even if the volume of the future liver remnant (FLR) increases, the liver’s function may not have fully recovered. Therefore, the timing of the second-stage surgery still needs to be determined based on the liver’s functional status. For patients with liver cirrhosis, ICG-R15 less than 40% after the first-stage surgery indicates sufficient FLR hypertrophy, and the second-stage surgery can be performed within one week. Another systematic review suggests that considering the surgical difficulty and complication rates, portal vein embolization (PVE) balances safety, effectiveness, and timeliness compared to ALPPS [10]. 

Charalel et al., conducted a systematic review comparing the outcomes of ALPPS and PVE. From the analysis of 21 PVE studies with mean percent FLR hypertrophy data and hepatectomy data, the random-effects pooled estimate of mean percent FLR hypertrophy was 30.9% with an average interval of 40.3 days (SD 26.3 days). In contrast, the analysis of four ALPPS studies revealed a random-effects pooled estimate of mean percent FLR hypertrophy of 54.9% over a mean interval of 11.1 days (SD 3.1 days) [10]. Another comparative study performed by Chan et al., indicated that ALPPS induced a much greater FLR hypertrophy than PVE (FLR volume gain by 48.8%, an additional 12.8% over 6 days). The increase in FLR volume was more significant in cases of chronic hepatitis compared to cirrhosis (52.7% vs. 32.5%) [9].

ALPPS could acquire the necessary liver volume more efficiently, but with a higher blood loss compared to PVE. Therefore, the choice between ALPPS or PVE as a transitional treatment should be based on the pros and cons of each. For patients with a future liver remnant (FLR) volume of 30–40% of the standard liver volume, PVE, which has smaller trauma and less blood loss, is sufficient to obtain the desired FLR volume. However, for patients with a higher risk of tumor progression and an FLR ratio of less than 40%, ALPPS should be considered. In general, ALPPS cannot completely replace the role of PVE. When selecting a transitional treatment strategy based on residual liver volume, clinical decisions should be made by considering the patient’s liver disease background, complication risks, and tumor progression. The goal is to maximize the patient’s survival benefits.

Furthermore, another multicenter retrospective study comparing liver venous deprivation (LVD) and ALPPS demonstrated that, with comparable complication and mortality rates, ALPPS exhibited faster hypertrophy of the residual liver and higher surgical resection rates. According to a global multicenter randomized controlled study, ALPPS surgery showed higher surgical resection rates (92% vs. 57%) compared to traditional two-stage hepatectomy (TSH) in patients with a future liver remnant (FLR) volume of less than 30% of the standard liver volume [11].

To reduce the complications and surgical risks associated with ALPPS and improve the second-stage resection rate, there have been continuous advancements in modified surgical techniques. Cillo et al. [12] reported a case of laparoscopic microwave ablation and portal vein ligation for staged hepatectomy (LAPS), where portal vein ligation was performed under laparoscopic assistance. Subsequently, ultrasound-guided microwave ablation was used to create an avascular trench along the pre-cut line of the liver, separating the residual liver from the embolized side. Microwave ablation ALPPS is a minimally invasive procedure that results in less trauma and faster recovery for patients compared to traditional ALPPS. Additionally, LAPS does not involve the transection of liver parenchyma, significantly reducing the risk of bile leakage and intraoperative bleeding. During the second surgery, the liver is opened along the ablation zone, improving the safety of the procedure.

Alvarez et al., performed partial ALPPS by dividing the portal vein of the diseased hemi-liver up to the middle hepatic vein, without extending the parenchymal transection further (up to the inferior vena cava) [13]. Compared to complete ALPPS, partial ALPPS results in comparable FLR hypertrophy and reduced morbidity [13,14]. A systematic review which included four studies and 124 patients showed the same result [15]. However, Chan et al., argues that complete ALPPS would induce more rapid FLR hypertrophy without increasing perioperative risk in chronic liver diseases [16]. Another meta-analysis performed subgroup analysis to evaluate patients with and without liver cirrhosis, and suggests that ALPPS had a better outcome in the cirrhotic group [17]. Petrowsky et al., contend that, though partial ALPPS perhaps induces slower hypertrophy when compared with complete ALPPS, it is sufficient to enable the rapid and beneficial second-stage operation leading to complete resection [14]. In summary, both partial and complete ALPPS procedures have similar effects in promoting remnant liver hypertrophy, but each of them has its advantages in different cohorts. Furthermore, additional research involving larger, homogeneous cohorts is required to further compare the outcomes of these two treatment approaches.

Some scholars have mentioned that in cases of insufficient liver hypertrophy, rescue ALPPS is performed as a salvage procedure [1]. Maulat et al., reported seven patients who underwent rescue ALPPS. Based on their experience, the prior use of PVE does not lead to more postoperative complications in rescue ALPPS procedures [18]. It can serve as an alternative after PVE failure and provides an opportunity for surgical resection in cases otherwise eligible only to palliative treatments [19]. Indeed, for those patients who have experienced PVE conversion failure, rescue ALPPS offers a glimmer of hope. However, ALPPS has been historically considered a high-risk procedure and is still in the process of ongoing development. Insufficient volume growth of FLR after PVE can be attributed to technical failures resulting from anomalous portal vein anatomy, as well as poor quality of liver parenchymal and impaired regenerative capacity due to prior chemotherapy. However, in the study by Maulat et al. [18], PVE did not add complications to subsequent ALPPS, continuing with a high-risk surgery for patients who have failed PVE conversion is a rather aggressive strategy. It is crucial to emphasize preoperative risk assessment and efficacy prediction for patients, involving comprehensive multidisciplinary teams to determine whether patients can tolerate the surgery and to manage the perioperative period effectively. Furthermore, more case data are needed to further clarify the survival benefits for patients undergoing salvage ALPPS. In addition, the success of salvage surgery is heavily dependent on the medical conditions of different centers and the expertise of the surgeons, which hampers the widespread practical use of rescue ALPPS.

Overall, PVE and ALPPS have their advantages and disadvantages, and the choice of procedure should be based on the patient’s specific tumor condition. Rescue ALPPS may serve as a salvage procedure for patients in whom PVE conversion has failed, providing an opportunity for complete resection. The surgical technique of ALPPS is still evolving and being refined, to further reduce complications and perioperative mortality. This provides an opportunity for an increasing number of unresectable patients to undergo surgical conversion.

## 3. Conversion Therapy for Oncologically uHCC: Making It Shrink

Currently, the indications for surgery in advanced HCC vary widely between countries and institutions. According to the AASLD Practice Guidelines and EASL Clinical Practice Guidelines, both of which were based on the BCLC staging system, surgical resection is not indicated in patients with advanced HCC. However, Chinese clinical practice guidelines for HCC do not exclude the indication of surgical resection on the basis of the tumor size or macroscopic vascular invasion. Even limited extrahepatic metastases may be candidates for surgical resection according to the Japanese clinical practice guideline. A similar expansion of the surgical indication has been proposed by the Hong Kong Liver Cancer Staging System and the Korean Liver Cancer Study Group, respectively. The main reasons for differences in treatment recommendations for advanced HCC between East and West may be different etiologies, different patient populations and different health economic conditions.

Although surgical treatment for advanced-stage liver cancer has shown suboptimal outcomes, most patients who undergo surgery do not exhibit significant survival benefits compared to medical treatment alone. However, research has found that conversion therapy can promote tumor regression and even lower the staging of liver cancer, thereby providing patients with opportunities for surgery and liver transplantation, leading to improved long-term survival outcomes. With the rapid advancements in interventional therapy and systemic anti-tumor drugs in recent years, various combination treatment approaches have achieved significant success in the management of advanced-stage liver cancer, driving progress in transitional treatment. The objective response rate (ORR) is an important indicator of tumor treatment, and partial response (PR) or complete response (CR) not only reflects sensitivity to the treatment regimen but also represents the potential for resection and surgical feasibility [20]. A global consensus should be reached on the indications for surgery in advanced HCC.

### 3.1. Combination of Systemic Anti-Tumor Drugs

Anti-tumor medications are the first choice for advanced HCC. The combination of atezolizumab and bevacizumab has been approved as the first-line treatment regimen for unresectable liver cancer patients. In the global multicenter Phase III IMbrave150 study [21], the median overall survival for the Atezolizumab plus Bevacizumab group was 19.2 months, with a progression-free survival of 6.9 months, significantly longer than the sorafenib group with 13.4 months and 4.3 months, respectively. The overall response rate reached 33.2%, demonstrating clear clinical benefits in the combination therapy group. In the global randomized double-blind Phase III LEAP-002 study [22], Lenvatinib plus Pembrolizumab showed a median overall survival of 21.2 months, a median progression-free survival of 8.2 months, and an overall response rate of 36.0% in patients with unresectable hepatocellular carcinoma. Combining CTLA-4 inhibition during the immune priming phase with PD-1/PD-L1 inhibition during the immune effector phase has emerged as a promising strategy in the field of cancer medicine [23]. In the global open-label phase III HIMALAYA study, a single high dose of the CTLA-4 antibody Tremelimumab in combination with the PD-L1 antibody Durvalumab yielded an ORR of 20.1% [24,25]. Currently, the combination of targeted therapies and immune checkpoint inhibitors is an important treatment approach for unresectable or advanced-stage liver cancer. It is also one of the main strategies for potential conversion treatment in potentially resectable liver cancer [2].

### 3.2. Interventional Therapy

Hepatic arterial infusion chemotherapy (HAIC) is the main local treatment method for patients with advanced-stage liver cancer. In recent years, FOLFOX-HAIC has demonstrated excellent efficacy in Chinese patients with advanced hepatocellular carcinoma (HCC). Some patients who undergo HAIC treatment experience a significant reduction in tumor burden or noticeable shrinkage of major vascular tumor thrombus, thus providing opportunities for conversion resection or ablation therapy. In an open-label Phase III clinical trial [26], advanced HCC patients treated with FOLFOX-HAIC achieved a median overall survival of 13.9 months and a median progression-free survival of 7.8 months, showing significant benefits compared to the sorafenib treatment group (8.9 and 4.3 months, respectively). Additionally, 15 patients (12.3%) achieved tumor downsizing and underwent curative resection or ablation, resulting in a median survival of 20.8 months and a one-year overall survival rate of 93.8%.

In another Phase III clinical trial conducted by Shi et al. [27], FOLFOX-HAIC demonstrated better outcomes than transarterial chemoembolization (TACE) in patients with tumors ≥7 cm in longest diameter, without major vascular invasion or extrahepatic metastasis. FOLFOX-HAIC showed a higher overall response rate (ORR) compared to TACE (46% vs. 18%), and due to tumor shrinkage and liver regeneration, 24% of patients underwent conversion resection surgery. The combination of loco-regional treatment also provides a new perspective for conversion therapy. A study from Sun Yat-sen University Cancer Center indicated that the combination of TACE and HAIC achieved an ORR of 65.9% (based on mRECIST criteria) and a conversion resection rate of 48.8% in patients with unresectable liver cancer, which was significantly higher than traditional TACE alone [28].

In addition, there is an active exploration of multi-modality conversion therapies combining systemic drugs with local treatments. Peng et al. [29] reported a Phase III clinical trial of TACE combined with Lenvatinib for advanced hepatocellular carcinoma. Compared to monotherapy with Lenvatinib, the combination regimen achieved a higher overall response rate (ORR) (54.1% vs. 24%, based on mRECIST criteria), with 15.3% of patients achieving downsizing and subsequent resection. A Phase II single-arm clinical trial investigated the therapeutic effect of HAIC in combination with Toripalimab and Lenvatinib in patients with advanced-stage liver cancer [30]. Among the patients in this trial, 86.1% had portal vein tumor thrombus and 27.8% had extrahepatic metastasis. The triple combination treatment yielded an ORR of 66.7%, with 15 patients (41.7%) achieving a partial response (PR) in the initial radiological evaluation, and 5 patients successfully undergoing surgical resection. The researchers pointed out that Lenvatinib and chemotherapy drugs can modulate the tumor immune microenvironment by stimulating antigenicity, thereby enhancing the efficacy of PD-1 inhibitors. The combination of targeted therapy and immunotherapy can also enhance the delivery of chemotherapy drugs by inhibiting angiogenesis and promoting vascular normalization (Table 1). 

Surgical resection is an important means for patients to achieve long-term survival after successful conversion, and the safety of surgery is an important assessment factor before conversion resection [31]. A retrospective study compared the complications and survival benefits between 320 patients who underwent conversion surgery after HAIC and patients who underwent direct surgery [32]. The HAIC surgery group consisted of 107 patients with initially unresectable advanced liver cancer. The HAIC surgery group showed a tendency toward postoperative liver dysfunction and intra-abdominal bleeding related to the number of HAIC treatments. Additionally, the recurrence-free survival (RFS) in the HAIC surgery group was comparable to that of the direct surgery group. Indeed, conversion therapy can bring hope for surgical resection to patients, but it can also cause damage to liver function and the overall physical condition of the patients. Therefore, it is essential to evaluate the general condition and liver function of patients during the process of conversion therapy and accurately determine the timing for surgical intervention.

In a separate study, Yttrium-90 radioembolization therapy resulted in a longer median overall survival compared to chemoembolization with drug-eluting beads in certain patients with unresectable HCC (30.2 months vs. 15.6 months, *p* = 0.006), and had a similar safety profile [33].

## 4. Perioperative Management of Patients after Conversion Therapy

### 4.1. Timing of Surgery

Surgical resection is an important tool for achieving long-term survival after conversion therapy. Choosing the appropriate timing of surgery requires consideration of both tumor response and surgical safety. Many scholars believe that patients with HCC that cannot be resected initially should accept surgery as soon as they meet the criteria of surgery after conversion therapy. However, one study showed that overall survival after resection in HCC patients was correlated with the objective response rate (ORR) of RECIST [34]. Patients with an objective response had longer postoperative OS. Progression-free survival as determined by RECIST correlated only modestly with OS, and the use of mRECIST or imRECIST did not improve this correlation. This view is corroborated by data from several RCT studies, where patients with objective responses have longer postoperative tumor-free survival [35]. In addition, the Chinese Expert Consensus on Conversion Therapy for Hepatocellular Carcinoma also concluded that for technically resectable CNLC stage IIb and IIIa HCC, surgery may provide better long-term survival when conversion therapy allows the tumor to achieve an objective response (shrinkage or downstaging) or to remain stable for a period of time (e.g., 3–4 months) [31]. A recent cohort study enrolling 101 patients, including 24 patients (23.8%) who underwent R0 resection, showed that a radiographic response to systemic therapy was more likely to result in curative resection. Hepatectomy after conversion therapy was independently associated with favourable overall survival. A pathological complete response to systemic therapy was associated with favourable recurrence-free survival after resection [36].

Considering the safety of surgery after conversion therapy, conversion therapy can cause damage to liver function at the same time as killing the tumor, thus increasing the risk of the surgery. Therefore, an interval is often needed between conversion therapy and surgery. This interval often varies depending on the type of preoperative treatment. For patients receiving systemic therapy, such as targeted drugs, it is not well established how long the drugs should be discontinued before surgery. From clinical experience, some guidelines recommend 1 week of preoperative drug discontinuation before surgery [31]. Notably, the anti-angiogenic effect of bevacizumab may lead to an increased risk of bleeding and decreased wound healing, thereby increasing the risk of adverse reactions. Therefore, bevacizumab should be discontinued for 8 weeks before surgery to ensure the safety of hepatectomy [37]. Immunotherapy is given every 2–3 weeks. It is generally considered not to have a large negative impact on surgical safety. In a study on the perioperative use of nivolumab and ipilimumab, surgery was performed 2–4 weeks after the end of the last drug cycle [38]. There is no conclusive evidence on the impact of the combination of targeted agents with ICIs on postoperative liver function and surgical safety. However, in a recent study, HCC patients treated with the combination of camrelizumab and apatinib, who discontinued apatinib 3 weeks before surgery and underwent surgery 4 days after the last immunotherapy treatment, showed manageable toxicity [35]. Notably, if adverse events occur during targeted therapy or immunotherapy, the surgery should be performed when the adverse events return to grade I or normal. In addition, a number of emerging biomarkers can be used to identify low-risk patients to reduce the number of conversion treatments, shorten the course of therapy or reduce the dose to protect liver function as well as reduce patient adverse events. TACE is the primary local treatment for advanced HCC, and it is recommended that the interval between the last TACE and surgery should be at least 4 weeks to reduce the impact on perioperative safety [39]. The impact of HAIC on the safety of hepatectomy is roughly similar to or less than that of TACE. In clinical practice, tumor evaluation is often performed 3–4 weeks after every 1-2 cycles of HAIC treatment to decide whether to proceed with surgery. Radiotherapy is also an important conversion therapy for advanced HCC, especially for patients with combined portal vein tumor thrombosis, and studies have shown that surgery 4 weeks after the end of radiotherapy results in good surgical outcomes and improves the long-term prognosis of patients [40].

### 4.2. Hepatectomy Techniques

For surgically unresectable patients, especially those without adequate preservation of liver function, transplantation is the best option if their tumor burden meets the criteria for transplantation. Because it can cure both the tumor and the underlying liver disease. In recent years, there have been a number of international extended liver transplant criteria compared to the Milan criteria, such as the University of California, San Francisco (UCSF) criteria and the Up-to-Seven criteria. Similarly, the Hangzhou criteria were proposed in China in 2008. Several clinical studies have confirmed that liver cancer patients who meet the Hangzhou criteria have satisfactory postoperative survival rates. The Hangzhou criteria can be further divided into two categories: Category A: tumor diameter ≤8 cm or tumor diameter >8 cm and AFP ≤100 μg/L; and Category B: tumor diameter >8 cm and AFP 100–400 μg/L. Recipients who meet the Hangzhou criteria in category A have a better prognosis [41]. However, due to organ shortage and long waiting times, TACE has become an acknowledged bridging treatment prior to liver transplantation. Furthermore, in the last decade, a new paradigm has emerged for early HCC occurring on compensated cirrhosis, which is the option of hepatectomy with salvage liver transplantation. Studies have shown comparable overall survival (OS) and disease-free survival (DFS) in patients treated with upfront and salvage liver transplantation [42].

Theoretically, anatomical resection could reduce the risk of recurrence after resection by impeding tumor cells from entering the remnant liver via portal blood flow. An RCT study showed that although the use of anatomic resection was associated with a significantly lower two-year local recurrence rate compared with non-anatomic resection (30% and 59%, respectively; *p* = 0.001), there was no significant difference in overall recurrence-free survival or overall survival between the two approaches [43]. Although there was no difference in perioperative or postoperative complication rates between the two approaches, anatomic resection was not commonly used due to the consideration of preserving as much normal liver tissue as possible. Especially with the rapid development of systemic therapeutic agents in recent years, the theoretical advantages of anatomical resection may need more evidence to be confirmed.

In addition, the status of the surgical margins, perioperative complications and pathological staging are all important factors that affect a patient’s prognosis. R0 resection is usually associated with longer RFS and OS. For patients whose resected tumor specimens show a pathological complete response (pCR), a shorter postoperative adjuvant treatment duration can be considered. In contrast, patients whose pathology suggests macrovascular or microvascular infiltration should be monitored more frequently with imaging and tumor markers or with more aggressive postoperative adjuvant therapy.

### 4.3. Adjuvant Therapy after Conversion Surgery

For patients with successful conversion resection, the expert consensus is that the original protocol may be selected for >6 months of adjuvant therapy if appropriate [31]. However, the choice of adjuvant treatment after R0 resection in successfully converted cases lacks sufficient data with high-level medical evidence. Some patients with recurrence may achieve clinical remission or disease stabilization after treatment with the original conversion regimen. Analysis of subsequent treatment options for relapsed patients may provide evidence for the choice of adjuvant therapy.

Recently, data from the IMbrave050 study revealed that atezolizumab in combination with bevacizumab showed a statistically significant improvement in recurrence-free survival (RFS) in patients with HCC after surgical resection or ablation, making it the first adjuvant treatment option to be confirmed in phase 3 clinical trial, which may provide a new rationale for postoperative adjuvant therapy [44].

Patients with advanced HCC who undergo surgery after conversion therapy tend to have a heavier tumor burden and a larger extent of the resection. Surgeons try to ensure that there will be enough residual liver in the future to avoid liver failure after resection, which leads to narrow margins. It is frequently associated with marginal recurrence [45]. A phase 2 clinical study suggests that intensity-modulated radiotherapy (IMRT) may improve disease-free survival (DFS) in patients undergoing narrow-margin hepatectomy [46]. For patients who have undergone conversion therapy and may still require adjuvant therapy, it needs more clinical evidence to demonstrate whether postoperative radiotherapy can improve the prognosis.

## 5. Conclusions and Prospects

Conversion therapy refers to a series of treatment measures aimed at converting patients who are not eligible for surgical treatment into candidates for surgical intervention. Unlike neoadjuvant therapy, conversion therapy aims to eliminate factors that make the tumor unresectable, while neoadjuvant therapy aims to improve prognosis and increase the rate of curative resection. However, both approaches share the common goal of controlling tumor progression, reducing tumor burden, and improving patient prognosis. For patients with technically unresectable CNLC stage I a, I b, and II a, the purpose of conversion therapy is to eliminate factors that hamper surgery performance. For patients with technically resectable but oncologically unresectable CNLC stage IIb, IIIa, and III b, the goal of conversion therapy is to improve the postoperative survival benefits for this group of patients [31]. By using systemic treatment methods, tumor burden is reduced to facilitate complete tumor removal and enhance surgical safety, or the tumor is down-staged for subsequent surgery, thereby further improving the patient’s survival benefits.

In recent years, the combination of local and targeted immunotherapy has become a research focus in the field of advanced-stage liver cancer. Increasing evidence shows that combination therapy has higher objective response rates and conversion-to-resection rates, with manageable adverse reactions, offering patients more hope for conversion. It is worth noting that various combination treatment strategies have achieved significant conversion outcomes, providing more opportunities for initially unresectable advanced-stage liver cancer to undergo surgery. However, the current targeted immunotherapy regimens in clinical research differ significantly, and patient inclusion criteria are inconsistent. As a result, there is currently no specific consensus on transitional treatment strategies. Further clinical trials and higher-level clinical evidence are needed to compare the pros and cons of different conversion strategies. Additionally, due to the heterogeneity of hepatocellular carcinoma and the various factors affecting individual prognosis and treatment sensitivity, further research is required to explore transitional approaches for different patient populations to achieve precise identification of conversion candidates, accurate selection of conversion strategies, and precise determination of the optimal timing for surgery. In the future, with the widespread application of interventional combined targeted immunotherapy and the active conduct of related clinical studies, transitional treatment can bring benefits to more patients with advanced-stage liver cancer.

## Figures and Tables

**Table 1:** Studies of interventional combination conversion therapy for patients with unresectable HCC.

Study	Design	Regimen (Patient No.)	ORR (mRECIST)	Surgical Rate	Outcomes
Lyu et al., 2022 [26]	Randomized, open label	Sorafenib vs. FOLFOX-HAIC (130)	FOLFOX-HAIC: 35.4%	11.5% (15/130)	mOS: 20.8 months estimated 1y OS rate: 93.8% (after curative treatment)
Li, Q.-J et al., 2022 [27]	Randomized, open label, multicenter	TACE (156) vs. FOLFOX-HAIC (159)	TACE: 33% FOLFOX-HAIC: 48%	TACE: 12% (18/156) FOLFOX-HAIC: 24% (38/159), 5/38 pCR	TACE mOS: 16.1 months FOLFOX-HAIC mOS: 23.1 months
Li, B. et al., 2022 [28]	Retrospective	TACE (42) vs. TACE-HAIC (41)	TACE: 16.7% TACE-HAIC: 65.9%	TACE: 9.5% (4/42) TACE-HAIC: 48.8% (20/41)	TACE mOS: 13.5 months TACE-HAIC: not reached
Peng, Z et al., 2022 [29]	Randomized, open label, multicenter	LEN (168) vs. LEN-TACE (170)	LEN: 25.0% LEN-TACE: 54.1%	LEN: 1.8% (3/168) LEN-TACE: 15.3% (26/170), 2/26 pCR	LEN mOS: 11.5 months LEN-TACE mOS: 17.8 months
Lai, Z et al., 2022 [30]	Single-arm single center	FOLFOX-HAIC + Lenvatinib + Toripalimab (36)	66.7%	13.9% (5/36)	6m-PFS rate: 80.6% mPFS: 10.4 months mOS: not reached

Abbreviations: HCC, Hepatocellular carcinoma; LEN, Lenvatinib; FOLFOX, Oxaliplatin, leucovorin, and 5-fluorouracil; HAIC, Hepatic arterial infusion chemotherapy; TACE, Transarterial Chemoembolization; pCR: Pathologic complete response.

## Data Availability

No new data were created or analyzed in this study. Data sharing is not applicable to this article.
